# Exposure to anesthetic gases in the operating rooms and assessment of non-carcinogenic risk among health care workers

**DOI:** 10.1016/j.toxrep.2023.06.007

**Published:** 2023-06-10

**Authors:** Fatemeh Kiani, Sahand Jorfi, Farhad Soltani, Saeed Ghanbari, Ramin Rezaee, Mohammad Javad Mohammadi

**Affiliations:** a Student Research Committee, Ahvaz Jundishapur University of Medical Sciences, Ahvaz, Iran; b Department of Environmental Health Engineering, School of Public Health and Environmental Technologies Research Center, Ahvaz Jundishapur University of Medical Sciences, Ahvaz, Iran; c Department of Anesthesiology, School of Medicine, Golestan Hospital, Ahvaz Jundishapur University of Medical Sciences, Ahvaz, Iran; d Department of Biostatistics and Epidemiology, School of Health, Ahvaz Jundishapur University of Medical Sciences, Ahvaz, Iran; e International UNESCO Center for Health-Related Basic Sciences and Human Nutrition, Faculty of Medicine, Mashhad University of Medical Sciences, Mashhad, Iran; f Department of Environmental Health, School of Public Health and Environmental Technologies Research Center (ETRC), Ahvaz Jundishapur University of Medical Sciences, Ahvaz, Iran; g Department of Environmental Health, School of Public Health and Air Pollution and Respiratory Diseases Research Center, Ahvaz Jundishapur University of Medical Sciences, Ahvaz, Iran; h Applied Biomedical Research Center, Mashhad University of Medical Sciences, Mashhad, Iran

**Keywords:** Anesthetic gas, Operating room air, Isoflurane, Sevoflurane, Health, Non-carcinogenic risk

## Abstract

Health care workers employed operating room in hospital and health centers are unavoidably exposed to inhaling toxic gases, including isoflurane and sevoflurane. Chronic contact with these gases increases the risk of spontaneous abortion, congenital anomalies and cancers. Risk assessment is an important tool in predicting the possible risk to personnel's health. Therefore, this study was conducted with the aim of determining the concentration of isoflurane and sevoflurane gas in the air of the operating room and estimating the non-carcinogenic risk caused by them. In this descriptive-cross-sectional study, according to the occupational method (OSHA 103), 23 samples (isoflurane and sevoflurane) were collected in the air of operating rooms of four selected hospitals in Ahvaz city by using SKC sampling pumps and sorbent tube (Anasorb 747). The samples were determined by used to gas chromatography with a flame ionization detector (GC/FID). Statistical analysis, including the Kruskal-Wallis test, was used to compare the average concentration of anesthetic gases, and the one-sample t-test was used to compare the average with the standard level. In all analyses, the significance level was 0.05, which was performed by SPSS version 22 software. Result of this study showed that the average concentration of isoflurane in private and general hospitals were 23.636 and 17.575 ppm, respectively. Also, the average level of sevoflurane were 1.58 and 7.804 ppm. According to the results the mean amount of anesthetic gases was within the range recommended by Iran's Occupational and Environmental Health Center and the permissible threshold limit provided by ACGIH. In addition, non-cancer risks from occupational exposure to isoflurane and sevoflurane in selected private and general hospitals were acceptable (HQ < 1). Although the results show that overall occupational exposure to anesthetic gases is less than acceptable but long-term exposure to anesthetic gases may endanger the health of operating room staffs. Therefore, it is recommended to implement some technical controls, including regular inspection of ventilation systems, the use of advanced ventilation systems with high cleaning power, continuous control of anesthesia devices in terms of leakage, and periodic training of related staff.

## Graphical Abstract

**Figure:**
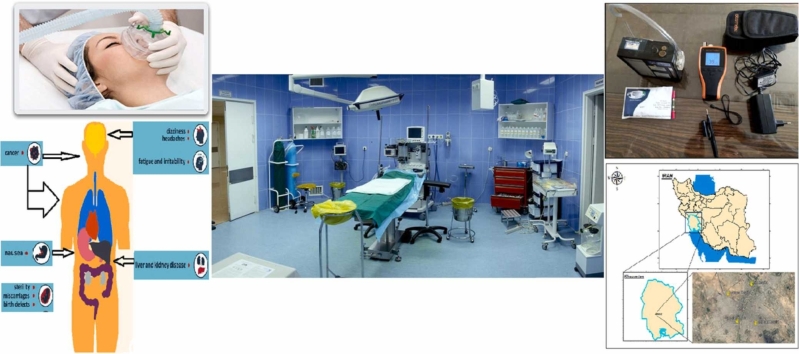

## 1 Introduction

Air is one of the most basic physiological needs of humans and other living beings. So, one of the most important things is to pay attention to the compounds in the air. Hospital operating rooms, which include operating rooms (ORs) or theaters, are among the most demanding areas of healthcare work. The air inside an OR contains various chemicals such as disinfectants, anesthetic gases, and sterilizing agents [14], [27], [30]. Inhalational anesthetics are used worldwide to maintain anesthetic in human and veterinary operating rooms [2], [37]. The most important anesthetic gases currently in use include nitrous oxide, halothane, enflurane, isoflurane, desflurane, and sevoflurane, and each one is used for different patients based on its advantages and disadvantages [28]. Anesthetic agents are the most important indoor air quality factors that increase the likelihood of serious complications among exposed healthcare workers and patients in operating rooms [37]. Occupational inhalation contact with these substances occurs when anesthetic drugs are used in operating rooms [18]. In 1977, it was estimated that there were 215,000 people who could have been put in danger by being exposed to the toxic gas. Regarding the level of danger of these chemical factors and the causes of workers' respiratory air pollution, we can refer to things such as the lack of proper air ventilation and scavenging systems and high concentrations of anesthetic gases in the air. Exhalation of patients in the post-anesthesia phase, common methods of anesthetic and gas leakage from cylinders and the anesthetic machine, duration of exposure, and type of equipment used to control inhalation concentration were mentioned [10], [19], [30], [39].

Isoflurane (CF3CHClOF2H) is the most common anesthetic gas used in the operating rooms of medical centers. Furthermore, isoflurane undergoes less hepatic biotransformation (0.2%) in comparison to halothane and sevoflurane. This anesthetic gas is more expensive than isoflurane. The minimum alveolar concentration relates to the anesthetic agent's potency. Isoflurane is not associated with cardiac dysrhythmias and is less metabolized than halothane and enflurane, so it has been replaced by halothane in some cases [36]. Sevoflurane (C4H3F7O) is a volatile fluorinated ether derivative widely used for general anesthesia. But due to contact with the carbon dioxide absorber that is present in anesthetic machines, it decomposes and produces a vinyl ether called compound A. If this compound is absorbed in large amounts, it can cause kidney damage [10].

Research on both animals and humans shows that long-term inhalation and exposure to anesthetic gases (isoflurane, sevoflurane) can caused health effects include reduced brain efficiency, reduced vision and hearing, reproductive system abnormalities, DNA damage, infertility, malignant hyperthermia (dominant autosomal genetic disorder in skeletal muscles), Glomerular Filtration Rate (GFR), irritation of the respiratory tract, liver and kidney diseases, megaloblastic anemia, increased prevalence of spontaneous abortions, headache, fatigue, nausea, vomiting, drowsiness, dizziness, irritation of the mouth and throat, decreased renal blood flow, congenital abnormalities, hepatitis, urinary output, weakening of the myocardial muscle (patients with myocardial contractile disorder caused by congestive heart failure are more sensitive to the cardiac debilitating effects of inhalation agents), the effect on the central nervous system and a higher incidence of cancer [1], [10], [17], [19], [21], [30], [34], [37], [39]. One of the most important findings reported from the study on women who work in the operating room is the higher than expected prevalence of abortion in them, and if there is no proper ventilation, this is a potential occupational hazard [39].

According to a number of studies, inhalational anesthetics like sevoflurane and isoflurane play a crucial role in carcinogenesis and decrease the immune response, which may not be desirable for cancer patients [12], [31]. Patients undergoing surgery for colon, breast, stomach, or rectal cancer who were given sevoflurane as an anesthetic had worse clinical outcomes than those given propofol, according to several retrospective studies [38], [4]. Isoflurane also increases the malignant activities of cancer cells, including proliferation and resistance to chemotherapy, through the upregulation of hypoxia-inducible factor 1 (HIF-1) expression in prostate cancer cells [32]. In order to minimize the risks associated with anesthetic gas exposure, the National Institute for Occupational Safety and Health (NIOSH) has set a limit of 2 ppm for exposure to halogenated anesthetic gases (without concurrent nitrous oxide use) for more than one hour [24]. In Iranian hospitals, anesthetic gases isoflurane and sevoflurane are mostly used, and contact with them may threaten the health of operating room personnel, so monitoring the amount of contact with these pollutants has become particularly important. Therefore, the aim of this study was measured the concentration of anesthetic gases (isoflurane and sevoflurane) used in the air of the operating rooms of government hospitals (Golestan and Taleghani) and private hospitals (Apadana and Aria) and evaluated the non-carcinogenic risk of anesthetic gases among health care workers in Ahvaz city.

## 2 Materials and methods

### 2.1 Characteristics of the study area

**Fig. 1:**
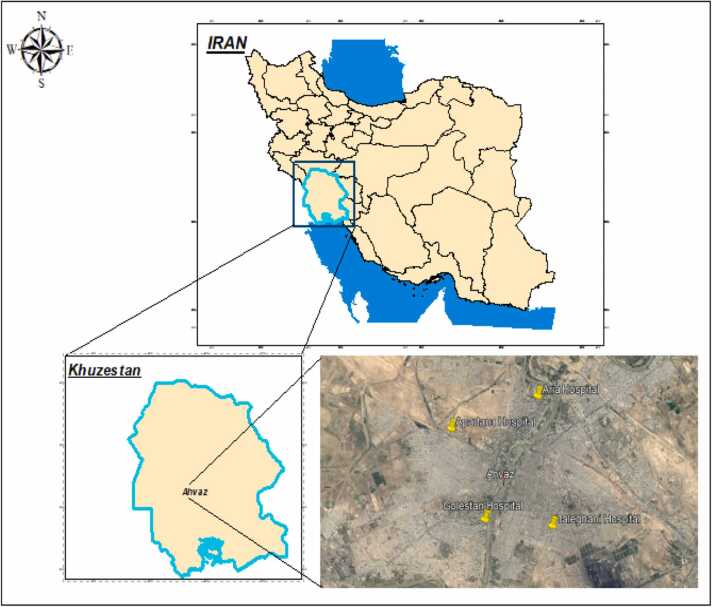
Geographical location of sampling points in the hospitals in Ahvaz City (Taleghani, Golestan, Aria, and Apadana).

This descriptive-cross-sectional study was conducted in one of the southwestern megacities of Iran during 2022–2023. Ahvaz is located at 31 degrees and 30 min of latitude north and 48 degrees and 65 min of longitude east, in the plains of Khuzestan, at a height of 12 m above sea level [11], [15], [25]. Among private and public hospitals, 2 hospitals were selected from each of them due to financial constraints. In this study, two private specialized hospitals (Aria and Apadana) with 358 beds and two general specialized hospitals (Taleghani and Golestan) with 619 beds were selected in Ahvaz city to measure the concentration of anesthetic gases (isoflurane and sevoflurane) in 2022 [22], [26], [33] (Fig. 1).

### 2.2 Eligibility criteria

In this descriptive-cross-sectional study, the first among hospitals affiliated and covered by the Ahvaz Jundishapur University of Medical Sciences 2 private and public hospitals were selected.

**Inclusion criteria:**

- Have active the operating rooms.
- Used to anesthetic gases (isoflurane and sevoflurane) in the operating rooms hospitals.
- Hospitals affiliated and covered by the Ahvaz Jundishapur University of Medical Sciences.
- Private and public hospitals.
- The study was conducted to investigate the anesthetic gases in the operating rooms and assessment of non-carcinogenic risk among health care workers.

**Excluded criteria if the operating rooms:**

- Inactive the operating rooms.
- Not Private and public hospitals.
- don’t used to anesthetic gases (isoflurane and sevoflurane) in the operating rooms hospitals.
- Not having an active ventilation system.

### 2.3 Sampling, standard preparation and analysis

**Fig. 2:**
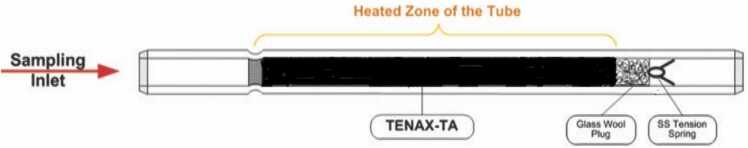
An image of a single Sampling-Bed Tube in this study (Tenax TA 250 mg).

23 samples (n = 23) were collected from the air of the general operating rooms of these 4 hospitals using a portable personal sampling pump (SKC pump) and glass packed with sections (70.140 mg) of Anasorb 747. With glass sampling tubes containing portions of Anasorb 747 (140/70 mg), samples were collected according to the guidelines of OSHA 103 (Fig. 2). A glass wool plug and two urethane foam plugs are used to hold the parts together. The end of the sampling tube was broken just prior to collection.

**Fig. 3:**
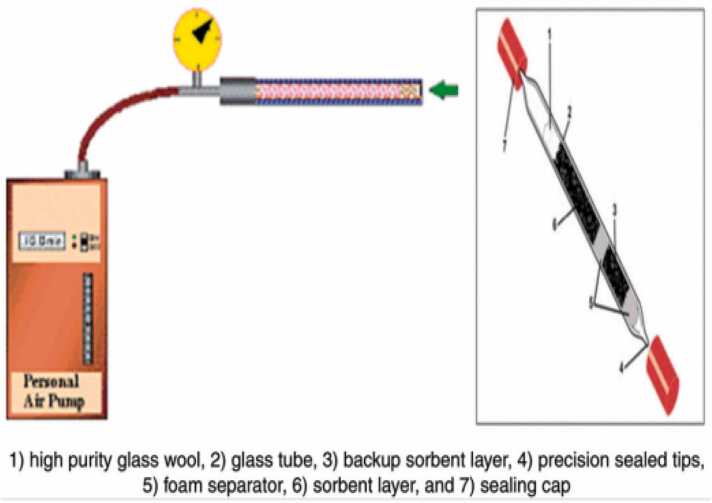
Sampling schematic using the SKC sampling device in this study.

Only fully trained people may collect samples in an operating room [28]. The SKC device was placed near the exhaust. Samples are desorbed with CS2 and examined using a flame ionization detector (FID) on a gas chromatograph (GC). 12 litres of air were sampled at a rate of 0.05 L/min. After a predetermined sampling duration, the sampling tube was promptly sealed with plastic end covers. Sampling schematic using the SKC sampling device in this study showed in Fig. 3.

### 2.4 Sample preparation

Concentrated stock standards of sevoflurane and isoflurane were prepared in toluene. Microliter amounts of concentrated stock standards were then injected into 2 mL vials containing 1.0 mL of desorption solvent, delivered from the same dispenser used for the desorption of samples. For example, to prepare an isoflurane target concentration standard, 10 mL of a stock solution containing 672 mg/mL isoflurane in toluene was injected into 1 mL of the desorption solvent. In the final step, the end plastic caps were removed, and each absorbent part was carefully moved to separate it. In the final step, the end caps were removed and each section of the adsorbent was carefully transferred to separate 2 mL vials.

After that, the glass tube, urethane foam plugs, and glass wool plugs were thrown out. Then, 1.0 mL of disposal solvent was added to each vial from the same dispenser that was used to prepare the standards. Then the vials were covered with polytetrafluoroethylene caps and shaken several times during the next 30 min. Finally, an FID was used to monitor effluent from sampling tubes containing only the 150 mg portion of Anasorb CMS or the 140 mg portion of Anasorb 747. The amount of analyte in each sampler was obtained from a suitable calibration curve in mg/m3, uncorrected for desorption efficiency [28], [3].

### 2.5 Assessment of carcinogenic risk

To assess the risk of carcinogenesis related to contact with soflurane and isoflurane gas, the average daily Intake (ADI) (mg.kg-1.day-1), through direct inhalation was calculated using the following formula (Eq. 1).

(1) ADI=(C×IR×ET×EF×ED)/(BW×AT)

In this formula, ADI is the average daily intake amount of the pollutant through breathing (mg.kg-1.day-1), and C is the concentration of the anesthetic gas (mg.m-3); IR is the inhalation rate in 8 working hours, which is equal to 20 (m3.day-1) for adults; ET is the exposure time, which is equal to 8 h in this study (hour.day-1); EF is exposure frequency (day.year-1); and ED is exposure duration (year); BW is the body weight (70 kg); AT is the averaging time (days) [28], [29].

### 2.6 Calculation of non-carcinogenic risk

In the next step, non-cancer risk was calculated using the following equation (Eq. 2).[20].

(2) Non-carcinogenic hazard Quotient (HQ) = ADI/RfC

In this regard, RfC (reference concentration) is equivalent to the reference value (mg.kg-1.day-1) determined by the US EPA. If HQ≤ 1, the significant non-carcinogenic risk does not threaten people, but if HQ> 1, the non-carcinogenic risk is high and unacceptable [16]. Table 1.

**Table 1:** Variables used to evaluate carcinogenic risk and non-carcinogenic risk[29], [8].

Variable	unit	Value
Concentration of anesthetic gase (C)	mg.m-3	Table 2
Inhalation rate (IR)	m3.day-1	20
Exposure time (ET)	hour.day-1	One shift (8 h)
EF)) Exposure frequency	day.year-1	300
(ED) Exposure duration	year	30
Body weight (BW)	Kg	70
Averaging time (AT)	Days	9000
Average Daily Intake (ADI)	mg.kg-1.day-1	Calculate
Reference concentration (RfC)	mg.kg-1.day-1	3.745 for sevoflurane and 7.546 for isoflurane

### 2.7 Statistical analysis of data

Statistical analysis consists of kruskal-wallis test to compare the mean of anesthesia gas (isoflurane and sevoflurane) in different hospitals and a one-sample t test to compare the mean with the level of standard. In all analyses, the significant level was 0.05, and they were done by SPSS software version 22.

## 3 Results and discussion

**Fig. 4:**
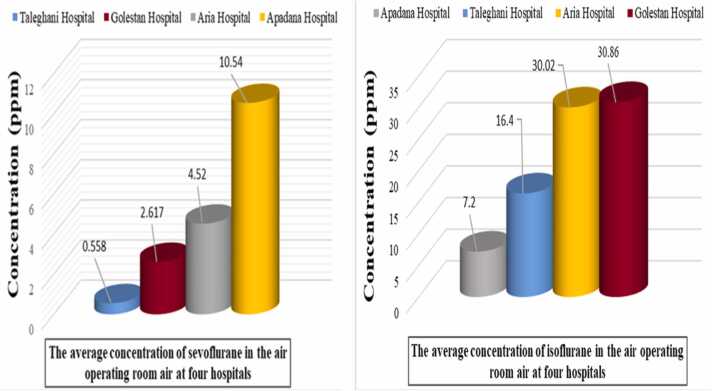
Comparison of average concentrations of anesthetic gases in selected hospitals.

In this study, the concentration of anesthetic gases (isoflurane and sevoflurane) in the air of operating rooms in four hospitals in Ahvaz, Iran, was investigated. According to the Kruskal-Wallis test results in Table 2 and Fig. 4, the average concentration of isoflurane in Apadana and Taleghani hospitals (respectively 7.2 ppm and 16.4 ppm) is significantly lower than others. The average concentration of sevoflurane in Taleghani hospital (0.558 ppm) is significantly lower than others, and in Apadana hospital (10.54 ppm) it is higher than others (Table 2 **and** Fig. 4). The maximum concentration of isoflurane is in Golestan Hospital (30.86 ppm), and the minimum is in Apadana (7.2 ppm). The highest concentration of sevoflurane is in Apadana hospital (10.54 ppm), and the lowest is in Taleghani (0.558 ppm) (Table 2 **and** Fig. 4).

**Table 2:** The average concentration of anesthetic gases measured in operating rooms of selected hospitals.

Variable	Hospital name	Mean (ppm)	Std. Deviation	Minimum	Maximum	Test statistic (P-value)
Isoflurane	Golestan	30.867	20.323	8.500	57.100	8.48 (0.037)
	Taleghani	16.400	22.403	1.900	58.300	
	Aria	30.020	21.757	11.100	64.300	
	Apadana	7.200	5.161	1.400	15.600	
	Total	20.735	20.068	1.400	64.300	
Sevoflurane	Golestan	2.617	1.441	0.300	4.300	11.55 (0.009)
	Taleghani	0.558	0.559	0.100	1.300	
	Aria	4.520	2.753	0.500	7.800	
	Apadana	10.540	11.474	0.500	31.200	
	Total	4.560	6.855	0.100	31.200

The values of occupational exposure limits and threshold limit are mentioned in Table 3. The average concentration of sevoflurane was within the threshold limit value (TLV) range recommended by Finland, Norway, and Iran's Environmental and Labor Health Center (Table 3).

**Table 3:** Occupational Exposure Limits and Threshold Limit Values [23], [38].

Anesthetic gas	OSHA	ACGIH TLV	Finland	Sweden	Iran
Isoflurane	None	50 ppm	10 ppm	10 ppm	50 ppm
Sevoflurane	None	None ppm	10 ppm	10 ppm	10 ppm

**Fig. 5:**
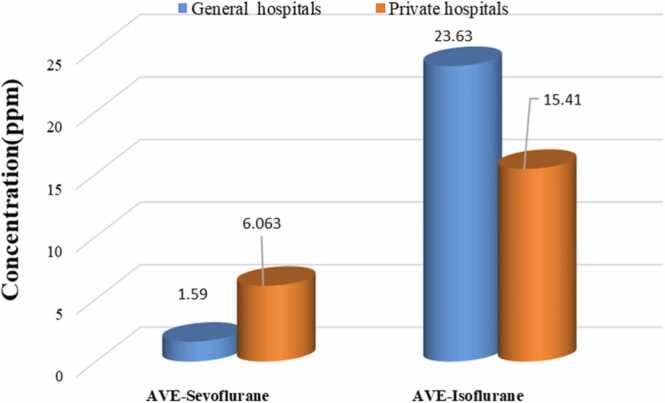
Comparison of the average concentration of sevoflurane in the air of operating rooms at four selected hospitals.

In general, in the comparison between two types of general and private hospitals in Ahvaz, the average concentration of isoflurane in the operating rooms of general hospitals (23.63 ppm) was higher than that of private hospitals (15.41 ppm) (Fig. 5), which can probably be due to the higher number of surgeries in general hospitals. Also, many surgeries in general hospitals are performed by residents who, due to their lack of experience and skill, take more time to perform surgery, which can be effective in increasing the concentration of anesthetics in the operating room [23]. On the other hand, the average concentration of sevoflurane in the air of operating rooms in private hospitals (6.063 ppm) was higher than that of general hospitals (1.59 ppm). There are several possible reasons for this difference. One of the most important is that sevoflurane is more expensive than isoflurane and is often only used in special cases. At the same time, it has unique properties such as a lack of respiratory irritation, which is especially useful for inducing anesthesia with the mask in children and adults, a non-pungent smell, and a faster change in the depth of anesthesia [8]. Among the causes of workers' respiratory air pollution, we can point out cases such as the lack of proper ventilation and scavenging systems, high concentrations of anesthetic gases in the exhaled air of patients in the post-anesthesia phase, common anesthesia methods, gas leakage from cylinders and anesthesia machines [37], [6].

Neisi et al. evaluation of anesthetic gasses of the indoor working room, Ahvaz, Iran. Agreeing to the comes about of Neisi et al. ponder, isoflurane concentrations in three primary instructive hospital ORs were 2.342, 2.15 and 2.04 ppm, individually. The mean level of isoflurane in three fundamental instructive hospital was more than suggested scales by worldwide organizations (2 ppm) and it sounds that presentation to this sum of gas would be the cause of wellbeing clutters for staff [28].

Afra et al. investigated to health risk assessment due to inhalation isoflurane in the operation room in Abadan, Iran. They reported that the Shahid Beheshti and Valiasr had the most noteworthy and the lowest amount of isoflurane. Concentration of isoflurane in Shahid Beheshti and Valiasr hospital were 2.436 and 2.129 ppm, individually [2].

**Fig. 6:**
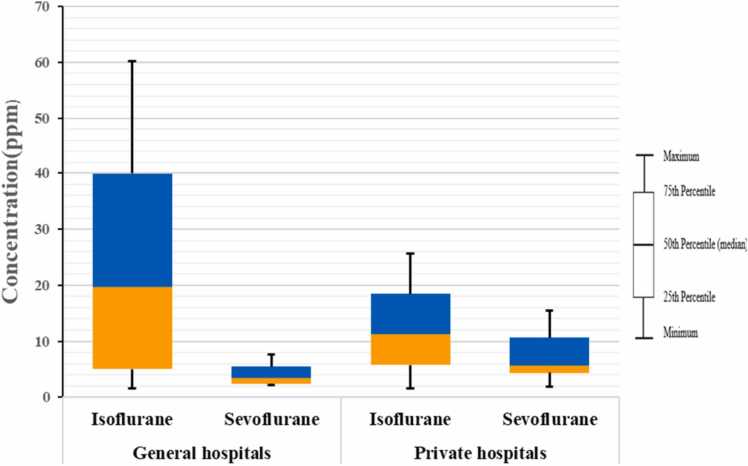
the average concentration of anesthetic gases in two types of private and general hospitals.

Table 4 shows the results of a one-sample t-test. It shows that the average concentration of isoflurane in the operating rooms of these hospitals is within the range recommended by the Iranian Center for Environmental and Labor Health and within the acceptable threshold limit set by ACGIH. Although the difference is not significant in some hospitals, the average is still lower. The biggest difference between the standard levels of isoflurane and sevoflurane is related to Apadana and Taleghani hospitals, respectively. It should be noted that the results of some samples (Table 2 and Fig. 6) showed that the concentration of isoflurane and sevoflurane in some work shifts was higher than the permissible threshold. It may be due to the lack of proper ventilation in the operating room or the lack of connection of the anesthesia machine to the ventilation valve, performing two operations at the same time in the same operating room, the difference in the type of surgery, or the number of surgeries during a work shift in the operating room. In line with this research, Damaso Fernandez-Ginez and colleagues investigated occupational exposure to halogenated anesthetics used for general anesthesia. The results showed that all tested sevoflurane concentrations were below the occupational safety agency exposure limits for Finland, Sweden, and Norway (10 ppm for an 8-hour TWA) [13]. In another study, Maryam Salehi et al. evaluated the occupational exposure of operating room personnel to isoflurane gas in Shahid Sadoughi Hospital, Yazd, Iran. The results showed that the level of contamination in the respiratory environment of the operating room personnel was higher than the permissible concentration recommended by the national institute for occupational safety & health (NIOSH standard) in 40.6% of all samples taken [35]. Martin and colleagues in a research study in Brazil stated that the average concentration for respiratory anesthetics in operating rooms is higher than the NIOSH standard [8].

**Table 4:** Investigating the relationship between the concentration of anesthetic gases and threshold limit values.

Hospital operating room (n = 23)	Anesthetic gas	Mean (ppm)	Std. Deviation	Std. Error Mean	Test statistic (P-value)
Golestan	Isoflurane	30.867	20.323	8.297	-2.306 (0.069)
	Sevoflurane	2.617	1.441	0.588	-12.55 (< 0.001))
Taleghani	Isoflurane	16.400	22.403	9.146	-3.674 (0.014)
	Sevoflurane	0.558	0.559	0.228	-41.38 (< 0.001)
Aria	Isoflurane	30.020	21.757	9.730	-2.053 (0.109)
	Sevoflurane	4.520	2.753	1.231	-4.45 (0.011)
Apadana	Isoflurane	7.200	5.161	2.107	-20.314(< 0.001)
	Sevoflurane	10.540	11.474	4.684	0.115 (0.913)
General	Isoflurane	23.633	21.747	6.278	-4.200 (0.001)
	Sevoflurane	1.588	1.497	0.432	-19.46 (< 0.001)
Private	Isoflurane	17.573	18.566	5.598	-5.793 (< 0.001)
	Sevoflurane	7.804	8.873	2.675	-0.82 (0.431)

Fig. 6 showed that the biggest difference between the standard levels of isoflurane and sevoflurane is related to Apadana and Taleghani hospitals, respectively. Also result showed that the concentration of isoflurane and sevoflurane in some work shifts was higher than the permissible threshold (Fig. 6).

### 3.1 Non-carcinogenic risk assessment

**Fig. 7:**
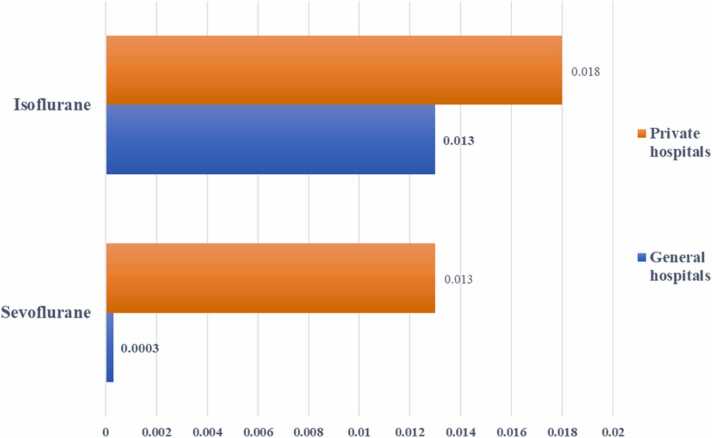
Non-carcinogenic risk assessment in selected private and general hospitals in Ahvaz city.

In this study, we evaluated the non-cancer risk of occupational exposure to anesthetic gases using the hazard quotient (HQ). It should be noted that in cases where HQ is above 1, there is a possibility of adverse health effects [9]. HQ was calculated from the average concentration of anesthetic gases (Table 2), and these data were used in the non-carcinogenic risk assessment. Data analysis showed that the HQ calculated for isoflurane and sevoflurane is below 1 in all hospitals (Fig. 7). In Iran and around the world, very few studies have been conducted to evaluate the concentration of sevoflurane and isoflurane vapors and to assess the non-carcinogenic risk of employee exposure to these vapors.

One of the studies was conducted by Fatemeh Tagvi and et al. in private and general hospitals in Sari, Iran. The results showed that the operating room staff's average exposure concentration to isoflurane was 17.6 ppm, with a maximum of 25.26 ppm and a minimum of 0.04 ppm. The concentration of isoflurane in the air of the operating room workers' breathing zone was higher than the recommended exposure limit recommended by NIOSH, but it was lower than the occupational exposure limits (OELs) suggested by the Iran Labor and Environment Health Center [7]. Omidi et al. evaluated the health risk of occupational exposure to isoflurane and sevoflurane in air samples from the respiratory areas of employees of two Iranian teaching hospitals. The results of their study showed that the measured concentrations of isoflurane and sevoflurane were lower than the standard of the National Institute of Occupational Safety and Health (2 ppm) for technicians and nurses but not for anesthesiologists and surgeons. In addition, the non-cancer risk estimate from isoflurane was higher than acceptable for anesthesiologists, but acceptable for other occupational categories [8]. Afra et al. investigated to health risk assessment due to inhalation isoflurane in the two teaching hospitals in Abadan, Iran [2]. They estimated to hazard index was under 1.0 and it sum appeared that no noteworthy hazard ascribed to presentation to level of isoflurane. They found that the concentration of isoflurane was higher than the NIOSH exposure limit, but the non-cancer risk was less than one [2]. In line with the present study, Nissi et al. operating rooms of three teaching hospitals. They measurement of the level of sevoflurane in three hospitals were more than the standard level (2 ppm) and exposure to this concentration of sevoflurane may well be threaten for HCW. The results showed that the non-cancer risks are lower than the permissible limit and are in accordance with the findings of the present study [27].

Similarly, in a study conducted in a Brazilian hospital, Brazo et al. discovered that the average residual concentration of respiratory anesthetics, including isoflurane, in operating rooms without a cleaning system is higher than the recommended limit of NIOSH (2 ppm) [5]. Jafari et al. estimations of isoflurane and sevoflurane in working room faculty [18]. The results of the study conducted by Jafari et al. in a hospital in Urmia, Iran, showed that the average concentration of isoflurane in the air of the operating room is about twice the amount in the air of the breathing zone of the employees and is higher than the NIOSH limit (0.5 ppm) [2]. Different reasons can be stated for the difference in the results of this study and the proposed studies, including the operating room ventilation system and the frequency of air changes per hour, the different sampling method, the difference in the amount of anesthetic drug used, the efficiency of the anesthesia machine cleaning system, etc. Another important point is that many discrepancies in studies may be due to differences in measurement methods. However, in the current research and the studies conducted by Jafari, Tagvi, and Dehghani, Nisi, and Afra, the method of sampling by activated charcoal tubes and analysis by gas chromatography, which is a time-consuming and expensive method, was used, which is more accurate than the direct method. Another reason can be the difference between the duration of sampling in the present study and the reviewed studies; most of them were sampled in a period of less than 1 h, whereas the sampling and analysis of the present study were performed in accordance with OSHA-approved standard procedures during a work shift. According to the results of this study and other research, the level of waste anesthetic gases in the air of operating rooms can exceed some permissible limits [9]. Therefore, operating room workers are in long-term contact with the remains of anesthetic gas concentrations, and this is a worrying issue due to chronic contact with small and continuous amounts of these anesthetics and their unpleasant effects on health. But if there is a scavenging system, modern anesthesia devices that leak less are used, the residual concentration of anesthetic vapors is constantly monitored, and employees are trained, they will have much less contact with these anesthetics.

## 4 Conclusion

This study investigated the occupational exposure to anesthetic gases (sevoflurane and isoflurane) and non-cancer risk in the operating room air of two private hospitals (Aria and Apadana) and two general hospitals (Taleghani and Golestan) in Ahvaz, Iran. Based on result of this study, the average concentration of anesthetic gases was within the range recommended by Iran's Environmental and Labor Health Center and the permissible threshold limit provided by ACGIH. However, the results showed that the concentration of isoflurane and sevoflurane in some samples was higher than the permissible threshold.

In addition, the non-cancer risks caused by occupational exposure to isoflurane and sevoflurane in selected private and general hospitals were acceptable (HQ < 1). Although the results show that overall occupational exposure to anesthetic gases was less than acceptable, long-term anesthetic gas exposure may endanger the health of operating room personnel. Therefore, it is recommended to implement some technical controls such as use of advanced ventilation systems with high cleaning power, continuous service and maintenance of existing devices, regular inspection of ventilation systems, Use of new anesthesia devices and systems, continuous control of anesthesia devices in terms of leakage, periodic examinations of employees and periodic training of related staff.

## Funding

This work was part of a funded at 10.13039/501100005001Ahvaz Jundishapur University of Medical Sciences (AJUMS), and the financial support of this study (IR.AJUMS.REC.1401.063) was provided by AJUMS.

## CRediT authorship contribution statement

FK, SJ, FS, SG-H, RR, and M-JM were principal investigators of the study and drafted the manuscript. M-JM, SJ, and FK were advisors of the study. FK, SJ, FS, SG-H, RR, and M-JM performed the statistical analysis. All authors contributed to the design and data analysis and assisted in the preparation of the final version of the manuscript. All authors read and approved the final version of the manuscript.

## Declaration of Competing Interest

The authors declare that they have no known competing financial interests or personal relationships that could have appeared to influence the work reported in this paper.

## Data Availability

No data was used for the research described in the article. The datasets generated during and/or analyzed during the current study are available from the corresponding author on reasonable request.
